# The relationship between dynapenia and vitamin D level in geriatric women with type 2 diabetes mellitus

**DOI:** 10.14744/nci.2021.28009

**Published:** 2022-02-11

**Authors:** Ridvan Sivritepe

**Affiliations:** Department of Internal Medicine, Istanbul Medipol University Faculty of Medicine Pendik Hospital, Istanbul, Turkey

**Keywords:** Dynapenia, geriatrics, type 2 diabetes mellitus, vitamin D

## Abstract

**Objective:**

In this study, we examined the possible relationship between dynapenia and vitamin D (VD) levels in geriatric women with type 2 diabetes mellitus (T2DM).

**Methods:**

One hundred and twenty-two geriatric female patients aged 65–80 years with a diagnosis of T2DM were included in this prospective study. Physical examinations of the patients were performed, and biochemical tests were analyzed. The muscle strength of the patients was measured with a hand dynamometer. Dynapenia was defined as low grip strength with normal skeletal muscle mass index. In muscle strength measurements, for female patients, over 20 kg was accepted as normal and below 20 kg as decreased muscle strength. Patients were separated into three groups as <10 ng/ml, 10–30 ng/ml, and >30 ng/ml according to VD levels; according to the status of dynapenia, they were divided into two groups as dynapenic and non-dynapenic. By comparing all these parameters between these groups, the relationship between VD level and dynapenia was evaluated. In statistical analysis, significance was accepted as p<0.05.

**Results:**

While 54 of the patients (44.3%) met the dynapenia criterion, 68 patients (55.7%) were non-dynapenic. Patients were first compared according to their dynapenia status. VD level was significantly lower in the dynapenic group (p<0.05). In the correlation analysis, a moderate positive correlation was found between muscle strength and VD (p=0.033, r: 0.23). The patients were then compared according to the VD groups. In the VD insufficient group, muscle strength (p=0.015), body mass index (p=0.025), systolic blood pressure (p<0.01), and glucose (p<0.01) were statistically significantly higher.

**Conclusion:**

In the present study, we found a considerable relationship between VD levels and dynapenia in geriatric women with T2DM.

**T**ype 2 diabetes mellitus (T2DM) ranks 4^th^–5^th^ among the causes of death in developing countries and therefore an important public health problem worldwide [1]. Today, more than 400 million people are known to have T2DM, and this number is estimated to reach 642 million by 2040 [1]. Turkey, the Vitamin D deficiency’s (VDD) prevalence is one of the countries with the highest [2]. It is estimated that more than 65% of the country’s population suffers from VDD, and this situation is reported to be higher in the geriatric population [2]. VDD has attracted great caution in recent years, as it improves the quality of life with its treatment and has an important role in health. Many studies have researched the relationship between vitamin D (VD) level and T2DM risk, and it has been determined that VDD itself is a risk factor for the development of T2DM [3–5]. In diabetic patients, VD is not only connected with calcium-phosphorus balance and bone metabolism but is also one of the hormones involved in the pathophysiology of various chronic illnesses such as metabolic syndrome, obesity, cancer, heart diseases, and dementia [6, 7]. However, recent evidence has shown that VDD may be related with malnutrition and musculoskeletal system diseases [8]. Dynapenia, a consequence of malnutrition, is defined as a decrease in muscle strength due to aging without a reduced muscle mass [9]. As we know that this decrease, which occurs independently of neurological or muscular diseases, impairs physical performance in patients and increases the risk of falls, resulting in mortality and morbidity [9]. Patients with T2DM have been shown to be candidates for dynapenia, and a decrease in muscle mass, strength, and performance has been considered a diabetic complication [10]. There is a study in the literature evaluating the relationship between dynapenia and VD levels, but there are no studies examining these two conditions in diabetic women [11]. Therefore, in the present study, we aimed to evaluate the interrelation between dynapenia and VD levels in women with T2DM.

## Materials and Methods

Our study is a single-center and cross-sectional study. Approval was obtained by the local ethics committee for the study (University of Health Sciences Umraniye Training and Research Hospital Clinical Research Ethics Committee Number: B101TKH434HGP001/69 Date: 11.03.2021), and it was carried out in accordance with the Helsinki Declaration principles. For the present study, all patients were informed about the purpose and procedure and written informed consent was obtained. For the power analysis of the study, reference number 11 was taken as reference [11]. Considering the correlation coefficient between dynapenia and VD being 0.99, the sample size per group was calculated as a minimum of 52, with a Type 1 error of 0.05 and the strength of the study being 80%. With a 20% loss, a total of 125 patients were incorporated in the study. 125 geriatric female patients aged between 65 and 80 years with a diagnosis of T2DM admitted to our internal medicine outpatient clinic were included in the study.

### Inclusion Criteria

• Between the ages of 65 and 80
• T2DM diagnosis according to American Diabetes Association criteria [12]
• Absence of acute diabetic problems such as diabetic ketoacidosis, hyperosmolar hyperglycaemic coma, or hypoglycemia in the past 3 months
• Having normal kidney and liver functions (Creatinine <1.1 mg/dl, aspartate transferase <34 U/L and alanine aminotransferase <55 U/L)
• A normal lifestyle.

**Highlight key points**

• The muscle strength of all diabetic and geriatric women with VDD should be measured.
• There is a clear relationship between VD levels and muscle strength in geriatric women with T2DM.
• Diabetic geriatric patients should be kept physically active to benefit from more sunlight, VD replacement should be initiated if necessary.

### Exclusion Criteria

• Under 65 or over 80
• Type 1 DM
• Hypo-hyperthyroidism or hyperparathyroidism
• Use of drugs that may change serum VD levels (such as VD, bisphosphonates, corticosteroid, anticonvulsant, and estrogen) or dietary supplements
• Body mass index (BMI) >30 kg/m^2^
• Presence of malignancy
• Cushing’s disease
• Addison’s disease
• Presence of sarcopenia
• The presence of quadriplegia, myopathy, or mobility due to stroke
• Severe cardiovascular disease
• Acute or chronic infection
• Diabetic neuropathy
• Major psychiatric illness.

Medical history was taken from all participants and physical examinations were performed. Diabetes duration, daily number of cigarettes smoked, and drug use of all participants was written down. Patients who smoked one pack of cigarettes a day were considered current smokers. Blood pressure was measured with an electronic device (Omron M2 Basic HEM-7121JE Arm Meter Digital Sphygmomanometer) in sitting position after a rest period of more than 5 min. Blood pressure and blood samples of the patients were taken between 08:00 and 10:00 h on an empty stomach.

### Evaluation of Metabolic Parameters

Biochemical blood tests of all patients were analyzed. Blood samples were taken from the patients, then they were analyzed in the same laboratory. Plasma glucose by the enzymatic test method, glycosylated hemoglobin by high-performance liquid chromatography method, calcium, phosphate, alanine transaminase, aspartate transaminase, high-density lipoprotein cholesterol, total cholesterol, and triglyceride concentration by enzymatic colorimetric test (Hitachi 747 autoanalyzer, German), creatinine by Jaffe` method, blood urea nitrogen by spectrophotometer, potassium, sodium, and chlorine level with ion-selective electrode analysis was measured with Architect plus device. The serum sample taken to measure the VD levels of the patients was centrifuged at 3500 rpm for 15 min and then measured by the electrochemiluminescence method. VD was measured using the Elecsys 2010, Roche Diagnostics, GmbH, Mannheim, Germany device with an intra and inter test coefficient of variation of 3.0% and 3.3%, respectively. The serum VD detection limit was 2 ng/ml. We know that VD level is affected by seasonal factors. Therefore, the study was carried out in January, February, and March. Patients were grouped according to their VD measurement levels as deficient (0–19.9 ng/ml), insufficient (20–29.9 ng/ml), and normal (30 ng/ml) [13].

### Definition of Type 2 Diabetes

We used American Diabetes Association criteria to detect the presence of T2DM in patients [12]. The patient was considered to have DM when the fasting blood glucose (8 h fasting) was ≥126 mg/dl and hemoglobin A1c (HbA1C) level 6.5% (48 mmol/mol).

### Evaluation of Anthropometric Measurements

The weights of the patients were measured after 12 h of fasting by removing shoes, socks, and heavy clothing, and using an electronic scale (Ekoter mechanical height measuring scales) that are calibrated every day. Their height was evaluated using a standard height gauge (Ekoter mechanical scale with height gauge). Bodyweight (kg)/height squared (m^2^) formula was used to calculate BMI.

### Evaluation of Muscle Strength

The right-left hand muscle strength of the patients was measured with a hydraulic dynamometer (12-0240 Baseline Hydraulic Hand Dynamometer 200 LB Standard W/Case). In the grip strength measurements, the patients were asked to hold the hand dynamometer in a standing upright position with the elbow straight at maximum capacity and squeeze it firmly for 3 s. This procedure was performed three times for each patient and the average of the dominant hand was calculated. Measurements of muscle strength were done before blood sampling so that the test was not affected. In the measurements, more than 20 normal and <20 were accepted as decreased muscle strength for female patients.

### Definition of Dynapenia

Patients’ values such as muscle mass (%), body fat ratio (%), metabolism/body fat, and lean body mass volumes were measured using a multi-frequency bioimpedance analysis (BIA) device (Fully automatic body bioimpedance analyzer, Tokyo, Japan). Skeletal muscle mass index (SMMI) was assessed as=([1/BIA resistance result×height^2^×0.401]+[age×–0.071]+[3.825×gender])+5.102. In the formula, height: centimeter, resistance: ohm, gender was considered female: 0 and age: year. The cut-off value for SMMI for women was accepted as 6.76 kg/m^2^: normal, between 5.76 and 6.75 kg/m^2^: moderate muscle mass reduction, 5.75: severe muscle mass reduction. According to Manini and Clark’s suggestion, dynapenia was defined as although the person has a normal SMMI index, a decrease in muscle grip strength [14].

### Statistical Analysis

Results were analyzed using MedCalc Statistical Software version 12.7.7 (MedCalc Software bvba, Ostend, Belgium; http://www.medcalc.org; 2013) was used. Descriptive terms (median, standard deviation, mean, maximum, and minimum) were used to describe continuous parameters in statistical analysis. Comparison of normally distributed continuous variables and independent was made with Student’s t-test. Comparison of two variables that were independent and not compatible with normal distribution was made using the Mann Whitney u test. Pearson correlation coefficient was calculated to determine the relationship between two normally distributed continuous variables, and for interpret relationship between two continuous variables that were not normally distributed Spearman’s rho correlation coefficient was used. Chi-square was used to estimate the relationship between categorical variables. For statistical analysis, Significance level was supposed as p<0.05. By comparing all parameters among the specified groups, the relationship between VD level and dynapenia in diabetic geriatric women was evaluated.

## Results

The study was conducted on 122 patients after the three patients who were included in the study left the study voluntarily. The mean age of the patients was 67.7 years, the mean muscle strength was 20 kg, the mean VD was 13.9 ng/ml, the mean HbA1c was 9.3%, and the mean duration of diabetes was 6.8 years. While 54 of the patients (44.3%) met the dynapenia criterion, 68 patients (55.7%) were non-dynapenia. The demographic data, biochemical and clinical parameters, and anthropometric measurements of the patients are shown in Table 1.

**Table 1.** Demographic data, anthropometric measurements, clinical and biochemical parameters of the patients (n=122)

Parameter	Mean	Median	SD	Minimum	Maximum
Age (years)	65.7	70	5.6	65	80
Muscle strength (kg)	20	31	4.2	2	44
BMI (kg/m^2^)	26.9	25.05	0.7	25.6	30
Waist circumference (cm)	96.7	100	8.4	89	128
SMMI index	7.32	7.2	1.01	6.34	9.5
Systolic blood pressure (mmHg)	124.6	130	12.5	100	160
Diastolic blood pressure (mmHg)	70.72	80	8.4	65	110
Vitamin D (ng/ml)	13.9	21.55	13	7.5	48.7
HBA1C (%)	9.3	8.875	4.2	5.5	14.9
Glucose (mg/dl)	195.5	143	66.4	60	563
Sodium (mEq/L)	138.8	139.5	6.3	131	147
Creatinine (mg/dl)	0.92	0.785	0.1	0.32	1.1
Aspartate transaminase (U/L)	21.3	21.5	2.1	10	34
Diabetes duration (years)	6.8	7.5	6.3	1	30
HDL (mg/dl)	21.52	23	3.2	18	53
LDL (mg/dl)	107.5	110	5.86	68	186
Cholesterol (mg/dl)	232.26	235	7.56	176	302
Potassium (mEq/L)	4.48	4.25	1	3.2	5.5
Blood urea nitrogen (mg/dL)	35.2	36.5	6.3	11	72.7
Alanine transaminase (U/L)	27.1	21	2.8	6	55

SD: Standard deviation; BMI: Body mass index; SMMI index: Skeletal muscle mass index; HBA1C: Hemoglobin A1c; HDL: High-density lipoprotein cholesterol; LDL: Low-density lipoprotein cholesterol.

VDD was found in 57 (46.7%) of the patients and VD insufficiency in 58 (47.5%), while VD was normal in only 7 (5.8%) patients. While 29.5% of the patients used only oral antidiabetic agents as antidiabetic agents, 38.2% were using only insulin. The percentage of patients using both oral antidiabetic and insulin was 32.3. 39.3% of the patients were non-smokers, 42.6% were active smokers and 18% were ex-smoker.

Patients were first compared as dynapenic and non-dynapenic (Table 2). VD level was found to be statistically significantly lower in the dynapenic group (p<0.05). Although BMI, waist circumference, duration of diabetes, and low-density lipoprotein (LDL) level were found to be lower in the dynapenic group, those were not at a statistically significant level. While diastolic blood pressure, systolic blood pressure, HbA1c, and glucose levels were higher in the dynapenic patient group, this ratio was not statistically significant.

**Table 2.** Comparison of dynapenic and non-dynapenic patients

	All patients (n=122)	Dynapenic group (n=54)	Non-dynapenic group (n=68)	p*
Age (years)	65.77±9.4	50.29±7.53	55.249.85	0.632
Muscle strength (kg)	20.01±16.5	10.92±19.5	27.23±12.2	0.01
BMI (kg/m^2^)	26.95±3.22	26.84±2.34	27.15±2.32	0.513
Waist circumference (cm)	96.63±8.2	95.71±7.22	96.62±8.39	0.63
SMMI index	7.32±1.01	7.21±1.03	7.34±1.06	0.68
Systolic blood pressure (mmHg)	138.58±21.5	139.05±25.43	136.11±23.47	0.988
Diastolic blood pressure (mmHg)	74.49±12.53	75.95±10.32	72.59±11.55	0.351
Vitamin D (ng/ml)	13.9±9.52	14.19±8.73	15.71±9.34	<0.05
HBA1C (%)	9.37±3.7	9.55±2.99	8.98±2.39	0.817
Glucose (mg/dl)	193.7±95.11	186±83.68	183.9±88.7	0.551
Sodium (mEq/L)	138.05±3.2	138.76±2.4	139.4±2.79	0.538
Creatinine (mg/dl)	0.99±0.97	0.9±0.16	0.92±0.25	0.532
Aspartate transaminase (U/L)	23.29±7.33	22.57±7.84	21.31±9.15	0.576
Diabetes duration (years)	9.52±6.53	5.05±3.58	7.33±5.79	0.218
HDL (mg/dl)	38.16±8.78	41.62±10.42	40±8.65	0.67
LDL (mg/dl)	148.32±69.42	127.14±33.79	137.24±57.39	0.591
Cholesterol (mg/dl)	206.19±53.13	210.76±78.96	200.42±41.55	0.872
Potassium (mEq/L)	4.49±0.43	4.36±0.49	4.52±0.41	0.367
Blood urea nitrogen (mg/dL)	36.37±11.7	31.87±9.47	34.99±9.58	0.162
Alanine transaminase (U/L)	29.95±19.8	29.86±14.19	28.7±23.59	0.23

BMI: Body mass index; SMMI index: Skeletal muscle mass index; HBA1C: Hemoglobin A1c; HDL: High-density lipoprotein cholesterol; LDL: Low-density lipoprotein cholesterol; *: Mann Whitney u test.

Patients were then compared according to VD levels (Table 3). Since there were not enough patients in the group with normal VD levels, evaluation was made between the group with insufficient and deficient VD. While muscle strength, BMI, SMMI index, and glucose levels were statistically significantly lower (respectively p: 0.015; p: 0.025; p<0.01) in the group with VDD compared to the group with insufficient levels, systolic blood pressure was found to be higher (p<0.01).

**Table 3.** Investigation of parameters according to vitamin D groups

Parameter	Vitamin D	n	Mean	Median	SD	Minimum	Maximum	p*
Age (years)	<10	57	68.5	67	10.01	65	80	0.041
	10–30	58	65.91	74	7.07	69	79	
	>30	7	68.9	68	11.2	65	80	
Muscle strength (kg)	<10	57	15	13	2.44	2	44	0.015
		10–30	58	25.52	24	2.83	10.1	44	
	>30	7	25.7	28	1.65	24	44	
BMI (kg/m^2^)	<10	57	24.25	27.45	2.44	24.6	32.2	0.025
	10–30	58	26.74	19.9	13.86	27.4	29.7	
	>30	7	25.4	25.4	1.74	25	28.4	
Waist circumference (cm)	<10	57	90	96	9.67	89	98	0.591
	10–30	58	96.79	97	5.72	97	128	
	>30	7	92.8	94	4.76	85	97	
SMMI index	<10	57	7.00	7.1	1.01	6.3	7.3	<0.05
	10–30	58	7.13	7.2	0.98	6.4	7.9	
	>30	7	7.89	7.4	1.2	7.4	9.5	
Systolic blood pressure (mmHg)	<10	57	148	140	18.5	110	160	<0.01
	10–30	58	127	130	22.7	110	160	
	>30	7	133	130	28.4	100	150	
Diastolic blood pressure (mmHg)	<10	57	76.74	80	12.83	60	110	0.078
	10–30	58	70.53	70	9.57	65	90	
	>30	7	73	75	8.36	65	85	
HBA1C (%)	<10	57	9.95	9	1.88	5.5	14.9	0.132
	10–30	58	9.81	7.7	1.09	7	12.8	
	>30	7	8.2	7.6	1.27	6.8	11.2	
Glucose (mg/dl)	<10	57	148	194	85.11	60	563	<0.01
	10–30	58	188	198	12.14	75	365	
	>30	7	124	152	64.68	69	173	
Sodium (mEq/L)	<10	57	140	139	3.02	131	147	0.525
	10–30	58	139.24	140	3.7	140	145	
	>30	7	140.2	141	3.11	137	144	
Creatinine (mg/dl)	<10	57	0.905	0.89	0.24	0.32	1.02	0.059
	10–30	58	0.95	0.615	0.13	0.52	1.1	
	>30	7	0.7	0.76	0.22	0.32	0.93	
Aspartate transaminase (U/L)	<10	57	30	19.5	7.77	10	34	0.445
	10–30	58	22.55	26.5	13.44	17	34	
	>30	7	18.4	16	6.87	23	28	
Diabetes duration (years)	<10	57	7.5	5	4.36	1	30	0.285
	10–30	58	7.98	3.5	3.54	1	20	
	>30	7	7.2	7	6.34	1	15	
HDL (mg/dl)	<10	57	39.19	38	8.32	18	53	0.524
	10–30	58	40.26	40	6.52	27	44	
	>30	7	44	42	10.01	29	42	
LDL (mg/dl)	<10	57	133.91	120.5	58.68	68	186	0.165
	10–30	58	156.27	134	80.84	72	175	
	>30	7	107.6	117	32.05	72	143	
Cholesterol (mg/dl)	<10	57	206	199	61	188	302	0.356
	10–30	58	206	204	38	182	302	
	>30	7	182	183	35.1	176	254	
Potassium (mEq/L)	<10	57	4.6	4.6	0.37	3.2	5.2	0.586
	10–30	58	4.45	4.5	0.14	4.4	5.5	
	>30	7	4.32	4.4	0.54	3.6	5	
Blood urea nitrogen (mg/dL)	<10	57	47.5	33.05	12.29	17	72.7	0.134
	10–30	58	35.21	29.05	4.31	26	55	
	>30	7	37.4	37.4	23.1	11	55	
Alanine transaminase (U/L)	<10	57	22	24.5	13.43	6	55	0.152
	10–30	58	30.19	22	11.31	14	55
	>30	7	16.6	19	6.73	8	23

SD: Standard deviation; BMI: Body mass index; SMMI index: Skeletal muscle mass index; HBA1C: Hemoglobin A1c; HDL: High-density lipoprotein cholesterol; LDL: Low-density lipoprotein cholesterol; *: Kruskal Wallis Test.

In the correlation analysis made between muscle strength and all other parameters; A moderate positive correlation between muscle strength and VD, SMMI index, LDL cholesterol, total cholesterol; A statistically significant negative correlation was found between muscle strength and age, diastolic blood pressure, creatinine. No relationship could be found with other parameters (Table 4).

**Table 4.** Correlation analysis between muscle strength and all parameters

	Muscle strength	
	Pearson correlation r*	p
Age (years)	–0.32	<0.001
BMI (kg/m^2^)	–0.048	0.42
Waist circumference (cm)	–0.143	0.754
Systolic blood pressure (mmHg)	–0.032	0.77
Diastolic blood pressure (mmHg)	–0.066	<0.001
SMMI index	0.86	<0.001
Vitamin D (ng/ml)	0.23	0.005
HBA1C (%)	0.085	0.168
Glucose (mg/dl)	–0.051	0.657
Sodium (mEq/L)	0.024	0.729
Creatinine (mg/dl)	–0.38	0.005
Aspartate transaminase (U/L)	–0.048	0.317
Diabetes duration (years)	–0.122	0.431
HDL (mg/dl)	–0.031	0.308
LDL (mg/dl)	0.41	<0.001
Cholesterol (mg/dl)	0.23	0.026
Potassium (mEq/L)	0.072	0.746
Blood urea nitrogen (mg/dL)	–0.085	0.98
Alanine transaminase (U/L)	0.024	0.367

BMI: Body mass index; SMMI index: Skeletal muscle mass index; HBA1C: Hemoglobin A1c; HDL: High-density lipoprotein cholesterol; LDL: Low-density lipoprotein cholesterol; *: Spearman Rho Coefficient.

## Discussion

In this study, we searched the possible relationship between dynapenia and VD, and we found a clear relationship between VD and dynapenia in this patient group. We found that while VD levels decreased in geriatric women with T2DM, patients’ muscle strength decreased, and VD decreased as muscle strength decreased. To the best of our knowledge, this is valuable because our study is the first study in this field in diabetic geriatric women.

VD has become an important field of study in recent years due to its role in the pathophysiology of many diseases, VD’s receptors (VDR) are discovered in many parts of the body and its positive effects on health by correcting its deficiency [15]. VDD is more common in geriatric individuals with increased fatness, decreased dietary VD intake, cutaneous synthesis of VD, and decreased sun benefit period [16]. Studies in the tropical and subtropical countries such as Turkey, Iran, India, Saudi Arabia, and China have shown that VDD has been seen in more individuals in recent years [2, 17, 18].

It is known that the prevalence of VDD is high in geriatric patients with T2DM, and this deficiency is associated with insulin resistance [4]. The prevalence of VDD was 46.7%, and the prevalence of VD insufficiency was 47.5% in diabetic women in our study. In an 11-year follow-up study, VDD has been shown to increase the risk of T2DM, regardless of family history [19]. In our study, we found that although it was not statistically significant, HbA1c levels were higher in the VD deficient group than in the VD deficient group. VDD is known to lower the intracellular calcium level, thereby reducing the level of insulin secretion and beta-cell function, resulting in disruption of the body’s response to glucose [5]. Previous evidence has provided that VDD causes diabetes-related problems such as peripheral neuropathy, nephropathy, retinopathy [20]. Our study showed that this deficiency can also contribute to the development of diabetic myopathy.

Several concepts have been put forward to indicate the severity of malnutrition symptoms and signs. One of them is dynapenia. The word meaning of “Dyna” is “power or strength,” “penia” means “poverty” [9]. Dynapenia means a decrease in muscle strength due to aging, even though the muscle mass is normal and without neurological or muscle diseases [14]. In studies conducted in the Far East, it has been found that the prevalence of dynapenia in women living in rural areas is between 14% and 27% [21–23]. In our study, we found the prevalence of dynapenia to be 44.3%. We think that the explanation for the relatively superior frequency of dynapenia in our study is that the patients included in the study resided in an urban area and were diabetic. Because inactive living in the urban area and diabetes is a big risk factor for dynapenia [14].

The number of studies evaluating the relationship between dynapenia and T2DM has been increasing in recent years. Many studies have shown that T2DM contributes to the reduction of muscle strength [24–26]. Although the mechanisms of the relationship between these two conditions are not fully savvy, it has been recommended that they may be related to insulin resistance and inflammatory cytokines. Sayer et al. [25] demonstrated in a study that there was a significant inverse relationship between muscle grip strength and blood glucose level and homeostasis model assessment-estimated insulin resistance. It is also claimed that accumulated advanced glycation products (AGE) in patients with T2DM can lead to dynapenia by contributing to the reduction of muscle strength [27]. In addition, the skeletal muscle cell plays an important role in absorbing and depleting glucose. It has been shown that myocyte functions are impaired due to the decrease in Type 4 glucose transporter protein levels in T2DM [28]. In all these studies, it has been shown that there is a closely relationship between T2DM and VDD and striated muscle functions. However, this prospective study demonstrated for the first time that VD levels were substantially lower in diabetic geriatric and dynapenic women. In the present study, VD level was found to be statistically significantly lower in dynapenic women. In addition, diastolic blood pressure, systolic blood pressure, HbA1c, and glucose levels were found to be higher in the dynapenic patient group. In diabetic women with VDD, we found that hand muscle strength and SMMI index were statistically significantly lower than the VD insufficient group. Furthermore, we established a positive association between VD and muscle strength.

In our study, there was no dissimilarity between dynapenic and non-dynapenic groups in terms of hyperglycemia therefore, we believe that the reason for the decrease in muscle strength in these patients is not hyperglycemia itself, but a direct VDD. At the same time, low muscle strength causes functional limitations and restricts activity, causing patients to be deprived of sufficient sunlight for VD synthesis. As a result, these two situations cause each other to develop and enter a vicious circle. We can explain with several mechanisms this significant relationship between low muscle strength and VD in our study. VDR have been tested in many tissues in the body, particularly in the beta cells of the pancreas and myocytes. VD is involved in the expression of more than 200 genes in these cells [29, 30]. Therefore, VD may directly affect striated muscle metabolism in patients with T2DM [31]. Having anti-inflammatory properties, VD inhibits cytokine-induced myocyte cell death in the inflammatory process. However, it increases the amount of muscle by increasing the effect of insulin known to be an anabolic hormone [32]. Therefore, in this case, we can conclude that sufficient VD level will protect muscle cells and prevent their progress to dynapenia. In addition to this, this decreases in muscle strength in patients may have occurred due to innervation and microenvironmental defect. Because it is known that VD affects neural stimulation [33]. Therefore, decrease in myocyte functions is an expected situation in VDD. Furthermore, it has been shown in studies that VDD causes advanced AGE accumulation. It has been claimed that these accumulated AGEs may cause sarcopenia by reducing muscle mass and strength in patients with T2DM [27]. In our study, VDD caused a decrease in muscle strength while muscle mass was normal, that is, without developing sarcopenia. This result makes a new contribution to the current literature. Recently, it has been discovered that dystrobrevin alpha, a member of the dystrophin-related protein complex, also acts as a VDR gene in skeletal muscle cells [34]. It is known that active VD synthesis from kidneys decreases due to aging [7]. Decreased VD cannot be able to stimulate the protein complex sufficiently. Therefore, loss of function may occur in myocytes.

In recent studies, it has been observed that the VDR is expressed to a lesser extent in damaged skeletal muscle [35, 36]. In a study of mice whose VDR was gradually destroyed, a decrease in muscle fiber size and muscle strength was observed [37]. In addition, in these mice, it was determined that the expression of sarcoplasmic reticulum calcium trasporter-ATPase and Calbindin, which are involved in the regulation of intracellular calcium concentration, was decreased [37]. It is evident that VD affects muscle function, improves size and mass, through related genes. However, the detailed mechanisms on this issue are still unclear.

It has been observed that the expression of forkhead box protein O1 (FOXO1), one of the transcription factors, is increased in some conditions such as malnutrition, inactivity, and cancer [38]. It has been shown that FOXO1 causes atrophy in myocyte cells through autophagy induction by activating the ubiquitin-proteasome system [39]. In a different study, it was found that VD suppresses the transcriptional activity of FOXO1. This may be one of the possible mechanisms of the link between dynapenia and VD in our study. However, it has been claimed that VD can stimulate mechanistic target of rapamycin complex 1 (mTORC1) signal in mammals and induce skeletal muscle hypertrophy associated with it. In a study established by Bass et al. [40] on rats, it was shown that excessive stimulation of the VDR caused an increase in mTORC1 and p-p70S6K protein, which caused hypertrophy in myocyte cells.

### Limitations of the Study

Our study had some limitations. First, our study was a cross-sectional study. Therefore, we could not establish a causal relationship between dynapenia and VDD. Second, the patients’ VD levels, anthropometric and muscle strength measurements were evaluated at a single time point. Third, our study was a single-center study, so our results may not be representative of all patients with T2DM. Fourth, there were factors in our study that we could not fix, such as the duration of sunlight exposure, diet, and inactivity. These factors can affect both VD levels and muscle function. Finally, the patients included in the study were middle-aged (65–80 years) women, so it is unclear whether our findings apply to women and men outside this age group. Despite these limitations, our study is important and valuable in raising awareness of dynapenic and VDD in geriatric diabetic women.

### Conclusion

We observed a significant relationship between VDD and dynapenia in geriatric women diagnosed with T2DM. We believe that this relationship is caused by VD by increasing regeneration and hypertrophy in myocyte cells, preventing apoptosis, and decreasing AGE accumulation. Our results may guide VD replacement studies for the prevention of dynapenia in women with geriatric T2DM.
